# Sir T. Lauder Brunton on Alcoholic Neuritis

**Published:** 1900-12-15

**Authors:** 


### SIR T. LAUDER BRUNTON ON ALCOHOLIC NEURITIS.

Writing on the subject of alcoholic neuritis, Sir T. Lauder Brunton [1] draws attention to two points in the diagnosis of the early stages of this condition, namely, the expression of the face and the condition of the pupil reflex. The face becomes mask-like and expressionless, the lips appear to move apart from the cheeks; but, what is sometimes still more extraordinary, the lips themselves may seem very mobile. The eyebrows and eyes may move in accordance with the lips, but a fixed and expressionless band stretches across the nose and cheeks between the eyes and lips, the skin upon the cheeks remaining motionless and unwrinkled while the lips, eyebrows, and forehead may be moving freely. The other point is the condition of the pupil reflex, which is just the converse of the Argyll-Robertson phenomenon. In a number of cases Sir Lauder Brunton has noticed that the reflex to light is rapid and extensive, whereas the contraction of the pupil on accommodation to a near object is slight and sluggish or entirely wanting. Indeed, in one or two cases he has noticed a dilatation instead of contraction on accommodation.

[1] British Medical Journal, December 1.

